# Onion (*Allium cepa* L.) and Drought: Current Situation and Perspectives

**DOI:** 10.1155/2024/6853932

**Published:** 2024-02-29

**Authors:** Oladé Charles Sansan, Vincent Ezin, Mathieu Anatole Tele Ayenan, Ifagbémi Bienvenue Chabi, Hubert Adoukonou-Sagbadja, Aliou Saïdou, Adam Ahanchede

**Affiliations:** ^1^Department of Crop Production, Faculty of Agricultural Sciences, University of Abomey-Calavi, Cotonou 01 BP 526, Benin; ^2^World Vegetable Center, West and Central Africa Coastal and Humid Regions, IITA-Benin Campus, 08 BP 0932 Tri Postal, Cotonou, Benin; ^3^Laboratory of Human Nutrition and Valorization of Food Bio-ingredients, Faculty of Agricultural Sciences, University of Abomey-Calavi, Cotonou 03 BP 2819, Benin; ^4^Laboratory of Genetic and Biotechnology, Faculty of Sciences and Technology, University of Abomey-Calavi, Cotonou BP 526, Benin

## Abstract

Onions (*Allium cepa* L.) are the second most commonly produced and consumed vegetable worldwide due to their economic, nutritional, and medicinal benefits. However, drought hinders vegetative growth, lowers yields and bulb quality, reduces photosynthetic activity, and alters the onion plant's metabolism. This review provides a summary of global research on the impact of drought on onions. It specifically seeks to shed light on aspects that remain unclear and generate research avenues. Relevant scientific articles were sourced from the AGORA database, Web of Science (WoS), and search engines such as Google Scholar, Scopus, MEDLINE/PubMed, and SCImago to achieve this objective. A total of 117 scientific articles and documents related to onion and drought were critically examined. The review revealed agromorphological, physiological, biochemical, and genomic studies depicting factors that contribute to drought tolerance in onion genotypes. However, there was little research on the physiological, biochemical, and genetic characteristics of drought tolerance in onions, which need to be deepened to establish its adaptation mechanisms. Understanding the mechanisms of onion response to water stress will contribute to fast-tracking the development of drought-tolerant genotypes and optimize onion production. Future research should be more focused on investigating onion drought tolerance mechanisms and structural and functional genomics and identifying genes responsible for onion drought tolerance.

## 1. Introduction

Onion (*Allium cepa* L.) is a bulbous vegetable from the Alliaceae family [1]. Onion production covered 215,934 hectares worldwide in 2021, yielding 4,562,530 tonnes [2]. Onions are the second most widely grown vegetable after tomatoes [2]. In West Africa, onions are the second most cultivated vegetable, with 955,617 tonnes produced on 42,911 hectares [2]. Onions have considerable economic potential for producing countries [3, 4]. The biochemical composition of onions makes them a valuable source of nutrition [5]. Onions are rich in bioactive elements such as flavonoids, phenolic compounds, organosulfur compounds, and polysaccharides, which are essential for human health [6, 7]. Onion production requires specific pedoclimatic conditions. Optimal onion vegetative growth requires a temperature range between 15 and 20°C, and bulb development requires a temperature range between 20 and 27°C. Onion production requires a soil pH between 6.0 and 7.0 and requires 350–550 mm of water [8, 9]. Regular irrigation is also necessary to maintain a high soil water content to ensure optimum yields [10]. However, in the context of climate change, global warming could lead to more frequent and intense heavy precipitation and droughts [11, 12]. A rise in both the frequency and intensity of extreme weather events, especially droughts, has been projected to worsen in the future [13]. Drought significantly affects the productivity of major crops, causing losses of up to 50% or complete crop failure [14, 15]. Onion, cultivated with shallow roots and water deficit [16], is especially susceptible to drought, posing high risks during its growth and bulbification phase [17]. Drought stress negatively impacts morphological parameters such as plant height, number of leaves, and leaf area, which results in significant yield losses of up to 65% in onions [1, 18].

Scientific evidence-based decision-making is crucial for enhancing onion production in water deficit situations. Onion and drought research has received significant attention in recent years. A search in Scopus and Web of Science using the keywords “Onion AND Drought” to produce 266 papers on Scopus (https://www.scopus.com/search/form.uri?display=basic#basic) gave an output of 234 papers that were published between 2008 and July 18, 2023. In Web of Science (https://www.webofscience.com/wos/woscc/basic-search), there were 289 papers found on the same subject, with 251 being published in the same timeframe. However, none of these papers reviewed recent developments in onion research regarding water stress. This emphasizes the importance of the timely review, which evaluates research on the mechanisms of onion response to water stress, the solutions developed for optimizing onion production under water stress, and the future outlook. This paper reviews the existing knowledge on onions and drought while proposing potential avenues for future research.

## 2. Methodology

To ensure the reliability and comprehensiveness of research on onions and drought, various search engines such as Google Scholar, AGORA, Scopus, MEDLINE/PubMed, SCImago, and Web of Science were consulted. Relevant articles, books, reports, and databases related to the onion, climate change, drought, and mitigation strategies were downloaded, reviewed, and analyzed for accuracy and validity. Any technical term abbreviations were explained on their first use. A search strategy using keywords in both French and English was exhaustively executed to maximize data collection. The keywords and/or key expressions utilized were as follows: “*Allium cepa* L.” “OR” “onion,” “drought stress” “OR” “water stress” “OR” “water deficit” alone or in combination “AND” with botanical, taxonomic origin, productivity/yield, economic and nutritional values, water requirements, climate change, morphological/physiological/biochemical/molecular parameters, genetic diversity, strategies, and improvement productivity. A total of 1,220 documents from 1957 to 2023 were downloaded, and duplicates were eliminated after preliminary sorting. Titles and/or abstracts were examined for relevance, language, publication type, field, and details to exclude those that are not relevant to this review. Finally, 139 articles from 1957 to 2023 were downloaded for detailed analysis and data extraction for this review.

## 3. Results

### 3.1. Botany, Taxonomy, and Origin of the Onion

Onion is a bulbous plant with a shortened stem called a plateau, which has cylindrical, hollow leaves on the upper part and adventitious roots emerging from the lower part [19, 20]. The leaves are arranged alternately, forming two rows facing each other [21]. The roots are numerous and whitish [22]. Onions have shallow, fasciculated roots that are slightly branched and extend 0.20–0.25 m deep into the soil [23, 24]. This limited root depth restricts water uptake in deep soils [25], thereby increasing their susceptibility to drought [26]. The flowers exhibit trimeric symmetry, with three sepals and three petals. Each flower contains six stamens and a three-loculated, superior ovary, each of which holds two large ovules. Pollen is released prior to the stigma's receptivity. This promotes cross-pollination among onion plants. Typically, each cluster yields between 100 and 1,500 seeds [27]. The onion bulb consists of tunics adorned with cataphylls or concentric scales that are thin, fleshy, and transparent [28]. Depending on the variety, the bulb may be yellow, red, white, or a combination of colors [21, 29]. Onion classification has been widely debated [30]. Onion was initially categorized in the Liliaceae family but was later reclassified in the Amaryllidaceae family because of its inflorescence structure. The use of molecular biology tools enabled the classification of onions in the Alliaceae family [30]. Numerous studies have shown that onion (*Allium cepa* L.) is a diploid monocotyledonous plant (2*n* = 16) belonging to the Asparagales order, Alliaceae family, Allieae tribe, and *Allium* genus [31–33]. The Liliaceae family comprises more than 3,700 species and over 250 genera [34–36]. Friesen et al. [37] reported that the *Allium* genus contains 780 species. Native to several Central Asian countries, including Turkey, Iran, Iraq, and Pakistan [31, 38], onion has become an important horticultural product in Africa, Europe, Asia, and America [39, 40]. Onion varieties have different colors (yellow, red, or white) and tastes [41, 42].

### 3.2. Economic and Nutritional Values of Onions

**Figure 1:**
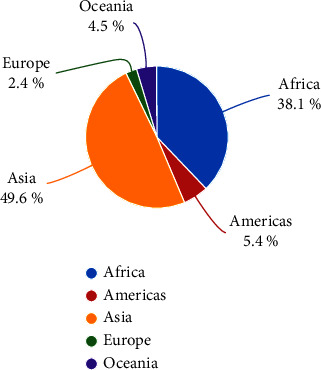
World onion production proportions by region in 2022 [2].

Onions hold immense economic and nutritional value, as reflected in their widespread cultivation. The dry onion was cultivated on 5967491 hectors and the global production was estimated to be 110616269.81 tons with a yield of 18.5365 tons/ha in 2022 [2]. Asia accounts for the majority of onion production, with 69.1%, followed by Africa (13.5%), Europe (8.6%), America (8.5%), and Oceania (0.3%) (Figure 1).

In 2022, among the top ten countries that produce onions globally, India leads with an average production of 31687000 tons annually, followed by China with 24542011.2 tons [2]. The eight additional major onion-producing nations, in descending order of production, are Egypt (3663943.34 tons), the United States of America (2918958 tons), Bangladesh (2517070 tons), Türkiye (2350000 tons), Pakistan (2062336 tons), Indonesia (1982360.22 tons), Iran (1900000 tons), and Algeria (1763117.95 tons). The top two onion-producing countries in Africa for 2022 are Egypt and Algeria [2].

The onions are grown commercially around the world not only because they contribute to overall economic growth but more importantly because they directly provide income to a large number of households in rural areas [43–45].

Nutritionally, onions play an important role in human health due to their high nutritional profile. Onion bulb is rich in crude oil, vitamins (A, B, C, and E), and minerals (sodium, calcium, potassium, and zinc) [7, 46]. Due to its vitamins (vitamin B, vitamin C, and provitamin A), minerals (potassium, sodium, zinc, iron, phosphorus, selenium, magnesium, manganese, and calcium), lipids, proteins, carbohydrates, essential oils, organic acids, and fiber content, onion is used as an energetic and protective food [47]. The outer layer of onions contains quercetin glucoside, quercetin aglycone, and kaempferol [48–50]. Quercetin inhibits the growth of many bacteria [51] and has the antifungal activity against *Aspergillus niger* and *Trichophyton rubrum* [52, 53]. In addition, the saponins and proteins present in onions have antimicrobial activity [54]. Several studies have reported the antiviral effect of flavonoids [55–57], causing lysis or inhibition of viral protein synthesis [58]. Onions are rich in natural antioxidants and polyphenols [3]. Owing to this nutritional and sensory value of valuable compounds, onion is greatly appreciated by consumers [59].

### 3.3. Water Requirements for Onion Production

Water requirements for onion production depend on several factors such as the season, variety, plant density, cultivation techniques, expected productivity, soil type, climate, and irrigation practices [60]. Onions are highly sensitive to water stress compared to other crops with deeper root systems [61]. For this reason, irrigation, especially during the bulb development phase, appears to be important for achieving higher yields [17, 62]. Onions require 350–550 mm of water for optimum yield [2]. Seasonal supply of 225–1040 mm of water to onion crops results in yields ranging from 10 to 77 t/ha [63]. Many studies have investigated the water requirements of onions in relation to productivity [64–66]. These have demonstrated that applying 225–602 mm of water to onion results in productivity ranging from 10 to 75 t/ha [64, 65]. Other studies have reported that applying 1040 mm per furrow and 602 mm per drip to onion crops gives an average yield of 59 t/ha and 77 t/ha, respectively [66]. These studies show that bulb yield increases are proportional to the amount of supplied water [16]. However, bulb yield decreases with irrigation above 602 mm and is almost zero when the amount of water is greater than 1,184.3 mm [67]. Therefore, water is a critical factor in onion production. These results will be useful in planning how to maximize onion yield through water supply in production areas.

**Figure 2:**
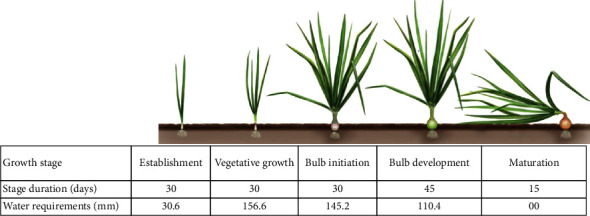
Onion growth stage and water requirements [68].

The water requirements for the different growth stages of onion are shown in Figure 2 according to reference [68].

### 3.4. Climate Change and Drought

Climate change is a 30-year shift in regional climatology, with the most adverse effects observed in Africa, Asia, and Central and South America [69, 70]. The direct impacts of climate change include regional changes in precipitation and the occurrence of extreme weather events (rising maximum and minimum temperatures, rising sea levels, and increased precipitation) [71]. Indirect consequences of climate change include water, food, and nutrition insecurity and loss of human life [70, 72]. Continued global warming could lead to an increase in the frequency and intensity of heavy precipitation events and droughts [11, 12]. This global warming will have increasingly dangerous consequences for natural environments and populations in all regions of the world [73]. Precipitation deficits are at the root of droughts and dry spells [74]. High temperature, high light intensity, and dry wind cause soil water loss through evaporation, which can exacerbate an existing water stress event [75]. Climate change will affect the entire globe in the coming years [76, 77]. Crop productivity, especially horticultural crops, will be significantly affected by severe water deficit stress due to the sensitivity of water availability to climate change [78]. West Africa's geographical location makes it one of the most vulnerable regions to climate change [79–82]. Considering the current and projected effects of climate change, the need to develop onion varieties with improved water use efficiency or adapted to water deficit cannot be overemphasized. Doing so requires a better understanding of the phenotypic, physiological, and molecular responses of the onion to drought stress.

## 4. Effects of Drought on Onions

### 4.1. Effects of Drought on the Vegetative and Reproductive Parameters of Onion

Onion is a crop that requires sufficient water for production. This shows that optimal soil moisture is necessary for onions to produce high yields [17]. However, a decrease in soil moisture leads to a reduction in onion plant height in the field [18]. Onion field and greenhouse experiments have shown that 20–45 days of drought negatively affects morphological parameters such as leaf number, length, width, and area, with a high rate of leaf senescence [1, 83, 84]. This proves that leaf senescence is a typical symptom during drought, which increases with stress severity in onions [85]. At the root level, drought reduces meristematic activity, which stops root elongation and bulbification of the root system [86]. Water deficit at the bulbification stage reduces bulb yield parameters in onion genotypes [83, 87]. Similarly, Dirirsa et al. [88] observed that water stress imposed at 50% of the maximum crop water requirement (ETc) had a significant effect on bulb size. Srinivasa Rao et al. [89] demonstrated in two onion cultivars (Arka Kalyan and Agrifound Dark Red) that drought stress induces a reduction in soil moisture, leading to a decrease in bulb fresh mass and consequently yield. Imposing a 15-day water stress on potted onion plants 30 days after transplanting results in a significant reduction in leaf area and bulb growth, with a 17–26% reduction in onion yield [90]. These parameters can enable breeders to classify onion genotypes into tolerant and sensitive categories and can therefore be used for screening and development of drought-tolerant varieties. Gedam et al. [85] conducted experiments on the selection of onion genotypes for drought tolerance and showed that tolerant onion genotypes performed better under drought with better agromorphological traits including significant leaf area, very high productivity, and less yield loss (<20%), whereas sensitive genotypes recorded large losses ranging from 60 to 70%. Moderately water stress-tolerant genotypes are characterized by taller plants with more leaves and relatively high yield losses estimated at 40–60%. These findings can guide irrigation scheduling to achieve optimal onion bulb production under water-stressed conditions.

### 4.2. Effect of Drought on Onion's Physiological Parameters

Water stress affects the physiological parameters of onion plants. Recent work has demonstrated that water stress severely altered the physiological parameters of onion genotypes when imposed during the developmental stage for 25 consecutive days [85, 91]. Photosynthetic pigments such as chlorophyll a, chlorophyll b, and carotenoids decreased in leaves when drought stress of 10–20 days was imposed on onion seedlings transplanted in pots and greenhouses [83, 92, 93]. This reduction in assimilative pigment levels during the growth phase causes a decrease in the photosynthetic activity, resulting in low organic matter synthesis in onion plants [84]. Several other works have demonstrated the effect of drought on chlorophyll in onion plants [91, 94]. These studies reported a reduction in chlorophyll content in onions under drought conditions. Continuous water deficit for 40 days further induces a reduction in the water status of onion leaves [1, 91, 95]. In addition, the somatic conductance of onion leaves decreases under water stress conditions [83, 95]. The decrease in gas exchange at the leaf level in onion cultivars is due to stomatal closure, which may be at the origin of the decrease in CO_2_ concentration. Under water deficit conditions, tolerant genotypes exhibit high values of physiological parameters such as chlorophyll a, chlorophyll b, carotenoid, and total chlorophyll, relative water content, membrane stability index, drought tolerance efficiency (>90%), membrane integrity, water use efficiency, and antioxidant enzyme activity than susceptible genotypes, which have the lowest values for the same parameters mentioned above [1, 85, 96]. The use of physiological traits has made it possible to group onion genotypes, especially according to drought tolerance. These physiological traits contributing to onion drought tolerance are likely to attract increasing interest, which needs to be judiciously used for the screening of tolerant genotypes. The behavior of physiological traits provides a better understanding of the morphological responses of onion genotypes to water stress. However, little research has been carried out on the effects of drought stress on photosynthesis and the photosynthetic electron transport chain in onion tree leaves. We suggest studies on alterations in leaf photosynthetic and the photosynthetic responses to drought stress in onion to identify tolerant genotypes.

### 4.3. Effect of Drought on Onion's Biochemical Parameters

Drought alters the biochemical parameters of onion genotypes. Thus, water stress decreases the synthesis of secondary metabolites such as phenols, flavonoids, tannins, and pyruvic acid [1, 91]. In contrast, drought causes an increase in proline, total sugars, total soluble solids, and hydrogen peroxide (H_2_O_2_) levels in cultivated onion genotypes [85, 91–93, 97]. The high level of hydrogen peroxide causes cell damage. Biochemical analyses showed that drought induces a very high accumulation of proline and malondialdehyde (MDA), including the increase of leaf antioxidant enzymes, especially catalase (CAT), superoxide dismutase (SOD), and ascorbate peroxidase (APX) in onion [84, 98]. The drought-induced accumulation of proline is explained by the activation of its synthesis and inhibition of its oxidation. The high accumulation of malondialdehyde is the consequence of the oxidative damage observed in the leaves. The high proline content in onions allows the plants to maintain their osmotic potential during water deficit stress and protects leaf membrane cells from damage. An increase in protein levels was observed by Vidyavani et al. [91], while Zheng et al. [87] and Ghodke et al. [1] reported a decrease in protein levels in water-stressed onion genotypes. This observed difference in protein levels could be partly due to the genotypic variability of the onion cultivars used and the intensity and duration of water stress. Drought-tolerant onion genotypes accumulate more proline and synthesize higher levels of antioxidant enzymes, especially catalase, which is one of the mechanisms of tolerance [1, 98]. Sensitive genotypes, on the other hand, have high levels of H_2_O_2_, which explains their sensitivity to drought [97]. These biochemical molecules associated with water stress tolerance in onions are traits of interest for identifying water stress tolerant and sensitive genotypes.

### 4.4. Effect of Drought on Onion's Molecular Parameters

**Figure 3:**
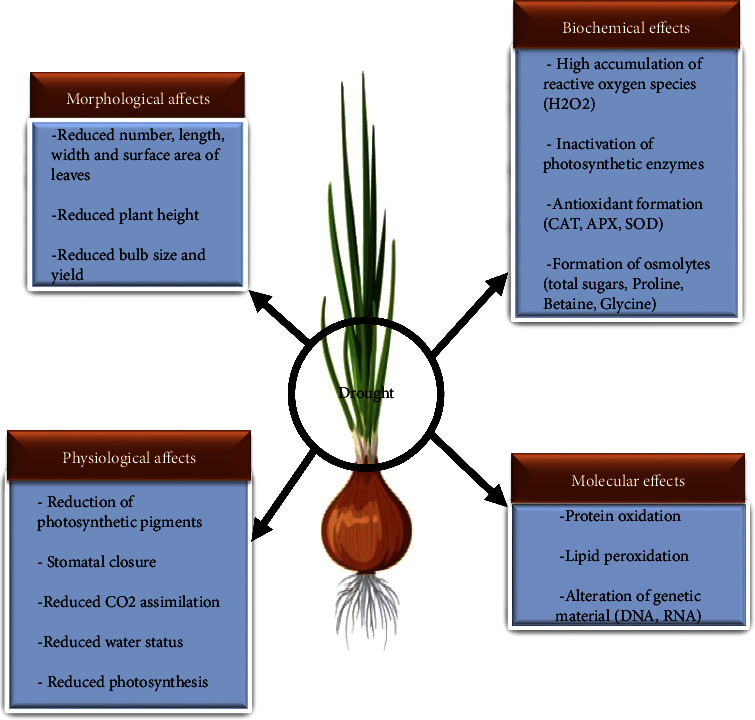
Impact of drought on different onion parameters.

Transcriptome sequencing of onion genotypes grown under water stress has been successfully used to reflect the different genes involved in onion response to drought. At the molecular level, under water stress conditions, drought-tolerant onion genotypes had a sharp increase in the expression of several genes such as those encoding transcription factors, cytochrome 450, membrane transporters, and those associated with carbohydrate metabolism and flavonoid biosynthesis [99]. These stress tolerance-related genes can be explored by researchers to screen for those potentially involved in onion drought tolerance. In addition, drought also induces several molecular markers in onions [100]. Recently, Chaudhry et al. [98] reported that drought induced an increased transcription of the catalase (CAT) gene but decreased the expression level of photosystem II genes. Exploring the genes involved in drought tolerance in onion and using them to screen for resistant onion genotypes is an effective approach. The genes that were highly expressed are promising candidate genes that could be used to develop new molecular markers. This would allow the integration of molecular and conventional breeding to speed up onion improvement programs. The results of the transcriptomic analyses could help scientists to better understand the molecular mechanisms of onion response to drought. However, limited information is available on molecular mechanisms that get altered in response to drought stress in onion. So, we will propose new studies on the transcriptomic analysis of drought response in onion using next-generation sequencing (NGS) technology to identify water stress key resistance genes.  Figure 3 shows the different effects of drought on onion.

### 4.5. Exploiting the Plant Genetic Resources of Onion

Onion is a highly diverse crop with 1,000 species worldwide [101]. Bağci et al. [102] reported the genetic diversity in a gene pool of 23 onion genetic resources consisting of 14 red and 9 white onions collected in different countries. This genetic variability can be used by breeders to identify or create new varieties with high performance and resistance to abiotic stresses, especially drought. For example, *F*1 hybrid onion varieties with parental genetic and metabolic traits essential for water stress tolerance were developed by crossing two onion species [103]. These *F*1 hybrids are mainly produced in temperate zones, and open-pollinated varieties are mainly grown in the subtropical and tropical climates of Asia and Africa [104, 105]. Numerous collection and characterization studies of onion accessions have been conducted in many countries to assess onions' phenotypic and genetic diversities. Mallor et al. [106] evaluated the morphological, physiological, and chemical parameters of 86 local onion accessions in Spain and found a wide variation in morphological traits and dry matter content. The morphological and molecular characterization work by Abdou et al. [20] revealed significant phenotypic and genotypic variabilities among Niger onion ecotypes. This phenotypic diversity relates to leaf length, width and leaf color, bulb weight, bulb shape and bulb color, and uniformity of bulb shape and color. Azimi et al. [107] carried out a morphological characterization of onion's genetic resources collected in Turkey and found that the skin of onion genotypes varied between white, reddish, and brownish-yellow in color and the bulbs had oval and flattened shapes. More recently, Gedam et al. [85] used multivariate analysis methods to determine the phenotypic and genetic diversities and then analyzed the relationships among onion genotypes under drought in India to classify them according to tolerance level.

Several molecular markers were also used to estimate the genetic diversity of onions. These include random amplified polymorphic DNA (RAPD) [108, 109], inter simple sequence repeat (ISSR) [110, 111], restriction fragment length polymorphism (RFLP) [112], amplified fragment length polymorphism (AFLP) [113, 114], and simple sequence repeat (SSR) [45, 115].

More works on the assessment of genetic diversity using morphological, physiological, and biochemical traits are necessary for better utilization of onion's genetic resources. The management of onion's genetic resources is becoming a necessity due to the overuse of improved varieties, which can reduce their genetic diversity and lead to genetic erosion [116]. In addition, knowledge of genetic variability is an asset for conserving material in a genetic repertoire and for easy accessibility and use of gene bank materials [117]. Sustainable use of onion genetic diversity requires *ex situ* conservation in gene banks and *in situ* conservation in farmers' fields [30].

Onion seeds are orthodox seeds and can be dried at a temperature of 15°C and a relative humidity of 10–15% before being stored in cold rooms at a temperature of 18°C or below to maintain their germination capacity [118]. Field gene banks are commonly used for the conservation of onion accessions through vegetative propagation, which allows the conservation of germplasm seeds for decades with 99% or 95% viability [119]. Approximately 12,740 onion accessions are conserved in gene banks worldwide [120]. Institutions such as the World Vegetable Center, Directorate of Onion and Garlic Research, European Cooperative Programme for Plant Genetic Resources, N.I. Vavilov Research Institute, National Institute of Agrobiological Sciences, Royal Botanic Gardens, US Department of Agriculture, and Warwick Crop Center are just a few of the international institutes conserving onion collections for sustainable use [121].

## 5. Strategies to Improve Onion Productivity under Water Stress

Mitigating the effects of water stress on onions requires a holistic approach including the development of drought-tolerant varieties, the use of growth regulators and plant extracts, the use of plant growth-promoting rhizobacteria (PGPR), and the development of production and irrigation systems that conserve soil moisture. We have already presented ongoing efforts to identify or breed water stress-tolerant onion varieties [1, 85]. Plant growth-promoting rhizobacteria (PGPR) in the rhizosphere colonize the roots of the plants, thus contributing to plant development, growth, yields, and water and mineral absorption of the plants. PGPRs have been used to enhance the resistance to the destructive effects of abiotic stress, especially water deficit tolerance in plants [122–125].

Recently, several other research papers have demonstrated that salicylic acid, potassium nitrate, abscisic acid, sodium benzoate, gibberellins, jasmonate, and polyamines are growth regulators used for plants under abiotic stress [126–129]. Wakchaure et al. [130] showed that the exogenous application of potassium nitrate (KNO_3_), thiourea (TU), salicylic acid (SA), gibberellic acid (GA), and sodium benzoate (SB) improved onion growth, yield, and quality under water stress. The application of salicylic acid to onion plants enables them to resist drought [131]. The application of hydrogel is an innovative solution that improves onion growth, development, physiological, and yield parameters during drought [132].

In addition, the root colonization of arbuscular mycorrhizal fungi improved leaf and root length, bulb diameter, bulb weight, and onion yield during drought [133]. This improvement results from the highly beneficial symbiotic relationship between onion plants and mycorrhizal fungi in which the mycorrhizal fungi mineralize the organic matter of the onion plants to induce their growth and development. In addition, the arbuscular mycorrhizal fungus (AM) *Glomus fasciculatum* increases nitrate reductase activity while enhancing nitrate uptake at the root level of onion plants under water-stressed conditions [134]. Inoculation of onion plants under drought conditions with arbuscular mycorrhizal fungi (*Funneliformis mosseae* and *Claroideoglomus etunicatum*) improved physiological and biochemical parameters such as total carotenoids, proline content, and soluble protein [135]. Mycorrhizal colonization keeps onion seedlings alive by improving their growth and development, thus promoting better onion production under stressful irrigation conditions [136]. In addition, the actinobacterium *Citricoccus zhacaiensis* is used to promote onion seed germination under drought conditions [137].

The use of both biochar and K-humate on onion plants during water deficit stress is an effective way to boost photosynthetic pigment content, mineral nutrition, growth, yield, and the enzymatic activity of antioxidant enzymes such as superoxide dismutase, catalase, and peroxidase, as well as the nutritional value of onions [95]. The application of humic acid to onion genotypes during irrigation deficits resulted in improved plant growth, increased soluble sugars and protein concentration, and enhancement of the enzymatic activities of foliar antioxidants [138].

To combat the impact of drought on onions, farmers can implement irrigation techniques. For instance, deficit irrigation (DI) applied during the initial growth phase (3 leaves) of onions, followed by an adequate watering, is a technique that boosts the water use efficiency of onion crops resulting in yield increase [139]. Shock et al. [16] and Enciso et al. [140] showed that implementing irrigation scheduling technology with soil moisture sensors, utilizing varying water potential ranges for onion cultivation, can result in substantial bulb yields with high water inputs.

### 5.1. Perspectives

Onion is a bulbous vegetable grown for human consumption in various parts of the world. The impact of drought on onions and their adaptation mechanisms has garnered scientific interest globally in recent years [1, 18, 85]. However, it is worth noting that the physiological mechanisms, along with molecular and metabolic architecture related to endurance, are yet to be fully understood. To address this knowledge gap, we suggest screening tolerant varieties utilizing agromorphological, physiological, biochemical, and genetic tools. In addition, it is crucial to conduct a transcriptomic analysis of onion genotypes to identify genes associated with drought tolerance. This process would facilitate the development of molecular markers and the selection of tolerant genotypes. Finally, it would be interesting to assess the beneficial impact of plant extracts, rhizobacteria, growth hormones, and specific agronomic practices on onion plants under drought conditions. These research avenues could bolster our understanding of the effect of drought on onions and aid in developing durable onion varieties and various technologies that can assuage the impact of water deficits for growers across the globe.

## 6. Conclusion

This review has discussed recent findings on the effects of water stress on onions. In short, drought has negative impacts on the agromorphological, physiological, biochemical, and genetic parameters of onions. In response to these effects, onion plants have developed various adaptation strategies. Exploiting the various parameters and tolerance mechanisms can be an asset in selecting resilient onion varieties from the genetic diversity available. The use of rhizobacteria, plant extracts, growth regulators, good agronomic practices, and appropriate irrigation systems have also proven to improve production in water deficient conditions. The morphophysiological, biochemical, and genetic responses, as well as their associated tolerance mechanisms and solution approaches to drought in onions, require further investigation. This research will aid in the generation of the necessary knowledge to fast-track the development of water-tolerant onion cultivars.

## Data Availability

The data used to support the findings of the study are included within the article.
